# Localized Therapeutic Approaches Based on Micro/Nanofibers for Cancer Treatment

**DOI:** 10.3390/molecules28073053

**Published:** 2023-03-29

**Authors:** Diana Alves, Joana C. Araújo, Raul Fangueiro, Diana P. Ferreira

**Affiliations:** Centre for Textile Science and Technology (2C2T), University of Minho, 4800 Guimarães, Portugal

**Keywords:** cancer, drug delivery system, nanofibers, nanoparticles, theragnostic

## Abstract

Cancer remains one of the most challenging health problems worldwide, and localized therapeutic approaches based on micro/nanofibers have shown potential for its treatment. Micro/nanofibers offer several advantages as a drug delivery system, such as high surface area, tunable pore size, and sustained release properties, which can improve drug efficacy and reduce side effects. In addition, functionalization of these fibers with nanoparticles can enhance their targeting and therapeutic capabilities. Localized delivery of drugs and/or other therapeutic agents via micro/nanofibers can also help to overcome the limitations of systemic administration, such as poor bioavailability and off-target effects. Several studies have shown promising results in preclinical models of cancer, including inhibition of tumor growth and improved survival rates. However, more research is needed to overcome technical and regulatory challenges to bring these approaches to clinical use. Localized therapeutic approaches based on micro/nanofibers hold great promise for the future of cancer treatment, providing a targeted, effective, and minimally invasive alternative to traditional treatments. The main focus of this review is to explore the current treatments utilizing micro/nanofibers, as well as localized drug delivery systems that rely on fibrous structures to deliver and release drugs for the treatment of cancer in a specific area.

## 1. Introduction

According to the World Health Organization (WHO), cancer is the second leading cause of death globally, with an estimated 9.6 million deaths in 2018. In 2020, the American Cancer Society (ACS) estimated that there would be approximately 1.8 million new cancer cases and 606,520 cancer deaths in the United States [1,2].

Cancer is a collection of diseases that can start in virtually any organ or tissue in the body when abnormal cells grow out of control, invade neighboring tissues, and/or spread to other organs. In the normal functioning of the human body, healthy cells grow and spread to generate new cells when the body requires them. As cells become damaged or age, they undergo apoptosis and are replaced by younger cells [3,4,5]. However, sometimes this orderly process is disrupted and abnormal or damaged cells (cancer cells) grow and multiply uncontrollably. This makes it difficult for the body to function properly, affecting the part of the body where cancer cells grow, leading to the appearance of tumors. Cancer cells can spread throughout the body using the circulatory and lymphatic systems, giving rise to metastases, which are the leading cause of death from cancer [3,4,5,6]. This disease develops due to multiple changes in your genes, which can have many possible causes, such as lifestyle habits, genes or exposure to cancer-causing agents [6].

There are several treatments available for cancer, such as surgery, chemotherapy, radiotherapy, immunotherapy, endocrine therapy, photodynamic therapy and hyperthermia therapy [7,8]. However, despite being a widely studied disease, the majority of treatments have many disadvantages, with many side effects, often highly serious for patients [9]. To circumvent these disadvantages and reach only the necessary target, drug delivery systems are increasingly being developed. These are technologies designed to deliver medicinal substances in a targeted and/or regulated manner. Several structures can be used as polymeric drug delivery systems, such as pharmacological films, hydrogels, wafers, sticks, microspheres, and fibers, among others. Fiber-based materials also offer several advantages for use in drug delivery applications. They are easy to fabricate, typically have high mechanical properties, provide a desirable drug release profile, and have a high surface area to volume ratio. To produce these intelligent systems, it is necessary to choose biocompatible materials linked to stimulus-responsive systems capable of controlling drug release [10,11]. Therefore, fibers composed of biocompatible and biodegradable polymers offer a low risk of inducing immune responses from the patient’s immune system, making them a favorable option for drug delivery applications. These fibers can be customized by adjusting their polymer composition, length, and cross-sectional radius. Additionally, the composition and morphology of the fibers can be tailored to meet the specific requirements of the application, resulting in low cytotoxicity, improved viability, and effective drug release [11].

The therapeutic properties of nanoparticles can also be used in cancer diagnostic techniques. Researchers managed to develop platforms for detecting and generating highly sensitive and specific images, showing great potential in detecting and diagnosing the disease, as well as monitoring the response to treatment. This monitoring is also called theranostic. Again, the fibers hold promise for theranostic drug delivery systems and may also incorporate imaging agents, allowing for both diagnosing and treating the disease [12].

Therefore, the development of localized drug delivery systems based on fibrous structures emerges as a promising strategy for the treatment of several types of cancer.

## 2. Diagnostic

The signs and symptoms of cancer are varied and numerous. They can also be similar to those of other diseases, such as infectious or autoimmune disorders, and can range in severity. In many cases, cancer does not present any signs or symptoms, especially in its early stages. Detecting cancer early requires both the individual and the healthcare team to be vigilant. Recognizing abnormal signs and symptoms and pursuing a proper diagnosis in a measured way offers the best chance of identifying cancer early and effectively managing it [13].

Cancer can be diagnosed through several methods, including physical exams, laboratory tests, imaging procedures, and biopsy [14]. During a physical exam, a healthcare provider may look for signs of cancer such as lumps, changes in the skin, or abnormalities in organs. The physical examination report for most cancers should provide detailed information on the tumor’s location, including the site and subsite, its extension to adjacent organs or structures, and the accessibility, palpability, and mobility of the lymph nodes. The report should also mention the probability of distant site involvement, such as the presence of organ enlargement, pleural effusion, ascites, or neurological symptoms. In the case of breast cancer, the physical examination should describe the precise location and size of the tumor mass, as well as the condition of the skin around the tumor, including any changes in color or texture and the mass’s attachment or fixation. The examination should encompass the entire axial and regional nodal area, including the supraclavicular nodes [15].

Laboratory tests may be performed on blood or other bodily fluids to look for abnormal levels of certain substances that may indicate cancer. Tests that measure the levels of certain substances in your body can indicate the presence of cancer [14]. However, abnormal results do not necessarily mean that cancer is present, and other tests such as biopsies and imaging are also used to make a diagnosis. It is important to note that laboratory results can vary among healthy individuals due to factors such as age, sex, race, and medical history. Normal results are often reported as a range based on the results of past tests from large groups of people. It is possible to have normal lab results and still have cancer, and likewise, abnormal results do not always indicate disease. Therefore, lab tests alone cannot provide a definitive diagnosis of cancer or any other illness. Imaging procedures, such as X-rays, CT scans, MRI, and PET scans, may be used to detect abnormalities in the body that may be indicative of cancer [13,16].

To confirm a cancer diagnosis, doctors often need to perform a biopsy, a procedure that involves removing a sample of abnormal tissue [14]. The tissue sample is then analyzed under a microscope and subjected to other tests by a pathologist to determine if cancer cells are present. Depending on the type and stage of cancer, one or more of these methods may be used to diagnose cancer. The findings are described in a pathology report that provides information about the diagnosis. Pathology reports are crucial for determining treatment options as they provide valuable information about the disease [13,16].

Prevention is often considered the best way to deal with cancer because it can help reduce the number of new cases and deaths from the disease. Cancer can be caused by a combination of genetic and environmental factors, and some of these factors can be modified or avoided. Overall, prevention measures can help reduce the burden of cancer on individuals, families, and society as a whole. By reducing the number of new cases and deaths from cancer, prevention measures can help save lives, reduce healthcare costs, and improve quality of life.

## 3. Cancer Therapies

The aim of cancer therapies is to considerably prolong and improve the patient’s quality of life and, if possible, cure [8]. Currently, there are several treatments available for cancer, such as surgery, chemotherapy, radiotherapy, immunotherapy, endocrine therapy, photodynamic therapy and hyperthermia therapy. These treatments change depending on the type of cancer being treated and whether it is at an advanced stage or not [7,8]. These therapies can be carried out individually, that is, only one treatment, or a combination of therapies can be used, trying to achieve the best possible results [7].

Surgery is the oldest method and is a procedure for removing the tumors from the patient’s body [17,18]. This method is not easy and cannot always be performed. In situations when it can be used, there is a chance that the cancer will not be entirely eradicated and, for that same reason, there is a risk that it will spread from its original position to other parts of the body, leading to metastasis. There are also secondary dangers such as bleeding, tissue and organ damage, pain and poor recovery of other body functions that are not well perceived by the patients in question [18].

Chemotherapy is a drug-based treatment to destroy cancer cells [17,19]. This treatment is used to reduce the tumor before surgery or to delay its growth until surgery [17,20]. Classical chemotherapeutic agents have their primary effect on macromolecular synthesis or function, contributing to cell death. This means that they interfere with the synthesis of DNA, RNA or proteins or with the proper functioning of the pre-formed molecule. When interference with macromolecular synthesis or function in the neoplastic cell population is large enough, a fraction of the cells dies. In other cases, chemotherapy may trigger differentiation, senescence or apoptosis. One of the problems with this treatment is that the drug is delivered through the bloodstream, reaching and affecting cells throughout the body, because it is a non-localized systemic therapy. Therefore, in selecting an effective drug, it is necessary to find an agent that has a marked inhibitory or growth-controlling effect on cancer cells and a minimal toxic effect on the host. Even so, there are several side effects in the body such as fatigue, nausea, loss of appetite and hair, and even blood clots [17,20]. Combination chemotherapy is a grouping of medications that is usually more effective in producing responses and prolonging life than medications used separately (monotherapy) and sequentially [17].

Radiation therapy is a treatment that uses ionizing radiation and deposits energy in the tissue cells it passes through to kill cancer cells or slow their growth. Radiation does not kill cancer cells immediately; it can take days or weeks of treatment before the DNA is damaged enough for the cancer cells to die, and there is a possibility that it can damage normal cells as well [8,17,21,22]. It is indicated to relieve symptoms (palliative treatment), shrink the tumor before surgery and kill remaining cancer cells when complete removal of the tumor is not possible, or after surgery to prevent recurrence [8,17,21]. Beyond that, the equipment is expensive, which leads to a high cost of the treatment, which is not always supported by the patients [22].

The immune system is responsible for helping the body fight infection and disease. Immunotherapy induces the immune system to fight cancer, through white blood cells, as well as organs and lymphatic tissues. This treatment has increased overall survival in several cancers at various stages of development, including metastatic disease. In contrast, some malignancies create immunosuppressive microenvironments characterized by high expression of immune checkpoint molecules, limited tumor antigen expression, and limited infiltration of circulating immune effector cells. These “cold” tumors, which are non-immunogenic and non-inflamed, do not respond well to immunotherapies and can successfully evade anticancer immune responses, leading to potential problems. The disadvantages of this treatment are that some drugs harm the organs and systems and it can take longer [23,24].

Endocrine therapy slows down or stops the proliferation of cancer cells that need hormones for their proliferation [25]. Although there are reports of successful cancer treatment with hormone therapy, this therapy is usually never used without the combination of another, as hormone therapy only attempts to extend “control” over the stage of cancer. In addition, side effects can significantly impair the patient’s daily life. The administration of hormones may result in organ damage and various side effects for the patient, including hot flashes, weight gain, muscle loss, breast swelling and tenderness, fatigue, and irritability. There is also an increased risk of anemia, cardiovascular disease (such as infarction), and metabolic syndrome, which can cause concern for those involved [26].

Photodynamic therapy involves the use of a photoactive molecule, light, and molecular oxygen present in tissues. When combined, these three compounds are capable of producing reactive oxygen species (ROS), which will induce target cell death [7,27,28]. Despite being a promising treatment, it has been used in a small number of patients, due to skin photosensitivity (the most common adverse effect) caused by systemically administered photosensitizers. Patients must avoid sunlight and strong artificial light for weeks, which is usually highly undesirable. Other limitations of photodynamic therapy are pain and decreased effectiveness for large or deep tumors due to difficulty in tissue penetration [29].

In hyperthermia, therapy body tissue is heated to temperatures between 40 and 43 °C to destroy cancer cells [30,31]. The hyperthermia procedure is based on the notion of subjecting body tissue to high temperatures in order to harm and kill cancer cells (by apoptosis) or to make cancer cells more specific to the effects of radiation and specific anticancer drugs [32]. There are several approaches used to apply this treatment: radiofrequency, microwave, water-filtered infra-red-A, ultrasound, and capacitive heating techniques. While hyperthermia therapy can elevate the intracellular temperature to the point of causing cell death, it has a significant limitation: cancerous and non-cancerous cells are often equally sensitive to heat. Hence, the most difficult aspect of hyperthermia is to maintain a high enough temperature in the tumor while keeping the surrounding normal tissues at a lower temperature to prevent damage to the healthy cells [33].

In fact, all existing therapies have advantages and disadvantages, which is why there is still no ideal solution for the treatment of cancer [9]. This has encouraged research and development of new strategies in order to find more effective and less invasive, painful and toxic treatments for patients. To overcome these drawbacks, drug delivery systems have been developed, which are technologies designed to deliver drugs in a targeted and/or regulated manner [34]. Various structures such as drug-eluting films, hydrogels, wafers, rods, and microspheres can be used as polymeric drug delivery systems. However, fibrous materials have several advantages for drug delivery applications [10,11,35,36]. In the next chapter, several examples of drug delivery systems using fibrous structures will be discussed.

## 4. Local and Systemic Drug Delivery Systems

**Figure 1:**
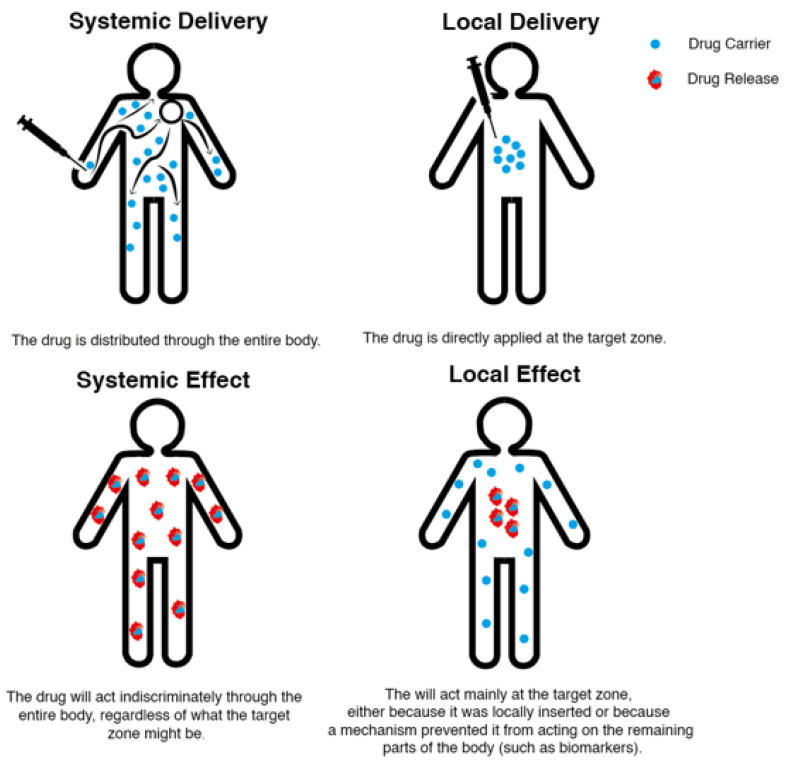
Illustration of the differences between systemic and local drug delivery systems and effects.

Drug delivery technologies are categorized according to the delivery method and effect site as systemic or local (Figure 1) [37]. This choice is critical, because selecting and regulating the drugs administered to a patient is crucial to an effective and comfortable treatment.

Systemic delivery methods are often adopted because they are easily administered, and since they are inserted through oral or intravenous routes, they are more tolerated by patients. Local drug delivery is by its nature more invasive. The medicine is frequently administered directly in the desired site through an injection. This method can have direct negative consequences if the target on body is highly sensible (for example the brain) and as such is frowned upon by patients [38].

Drugs that have a systemic effect are generally administered through systemic delivery methods, distributing the substance throughout the body, regardless of how small the target part might be [37,39]. Since the medicine acts indiscriminately in every part of the body, it can lead to harsh side-effects, particularly with anticancer agents. Furthermore, repeated dosage of the chosen substance is necessary to maintain an adequate concentration. Nonetheless, this leads to a concentration oscillation between doses, originating peaks and falls in each administration cycle. In some situations, this will result in a seesaw effect in which the minimum value is so low, it does not give any therapeutic effect, and the maximum value is so high, that several undesirable side-effects arise [40].

Considering the high costs, high risk, and the long time associated with the development and research of novel medication, a significant effort is being put into changing its effect [41]. Medications that have a local effect contrast with the previously described drugs because they are intended to act predominantly on a specific part of the body. Even if this localization is not perfect, it yields far more controlled results than the systemic effect, leading to an improved efficacy and reduced toxicity in off-target sites. This not only obliges a safer treatment, but in some cases might even allow a higher dosage administration of the desired drug [37]. This direction breathes new life into drugs that have already been developed, and becomes a safer research path to be taken by pharmaceutical companies overall. The synthesis method is known, market approval has been cleared, and clinical trials have already been successfully performed [42].

Drugs with a local effect can be administered systematically and locally. If the drug is administered systematically it needs to have a trigger for the release or a have a method to accumulate the particles in the desired site. In the case of cancer, this is typically done by placing a biomarker at the tumor cells, causing the drug carriers to gather at the intended local site [37]. However, since these particles will spread through the body, they need to have a smaller size (ideally less than 400 nm) to avoid any emboli in the blood stream [43] and prevent further complications in blood vessels [37]. The benefit of having a local delivery system is that a drug’s therapeutic concentration can be upheld for a longer duration without recurrent dosage, reducing drug under/overdosage concerns and delivering it to the required location [40]. Contrary to the previous method, this makes it easier to design the system and favors the usage of larger particles [37]. These are designed as drug-eluting films, hydrogels, fibrous structures, wafers, rods, and microspheres [44,45,46,47].

There is a growing recognition that success in drug delivery systems focuses to-wards developing increasingly compact devices and agents [48]. Over the past few decades, the utilization of nanotechnology has become widespread. Nanoparticle drug delivery is now regarded as a promising approach to cancer treatment, owing to the drug-loaded nanoparticles’ high loading capacity, reduced toxicity, stability, efficacy, specificity, and tolerability when compared to conventional chemotherapy drugs. Nanoparticles loaded with anticancer drugs have the potential to deliver drugs to tumors during cancer treatments, either actively or passively. One of the benefits of using nanoparticles is their ability to be produced in various small sizes, as well as being composed of different materials, including lipids (e.g., liposomes, solid lipid nanoparticles), polymers (e.g., polymeric nanoparticles), and inorganic substances (e.g., gold nanoparticles). Liposomes, micelles, polymeric nanoparticles, solid lipid nanoparticles, and gold nanoparticles are commonly used in cancer treatment among the different types of nanoparticles available [49].

### 4.1. Fibrous Structures as Localized Drug Delivery Systems for Cancer Treatment

Fibers (micro and nanofibers) can create very promising material structures for localized drug delivery systems, especially regarding cancer treatment. 

In order to avoid strong immune responses from the patient’s immune system, these structures can biomimic other biodegradable polymers with low immunogenicity. Furthermore, adjustments can be made to their structural integrity by changing the polymers used to make them, their length and their cross-sectional radius, finding the best composition and morphology for the application required [47].

Common strategies to improve the fiber’s performance include mixing synthetic and natural polymers, harnessing the properties of each polymer in order to create a superior arrangement. Many polymers and polymer mixtures have been studied for fiber structure production. As such, several studies were performed with different polymers, analyzing how several of these structures can be used in drug delivery systems for cancer therapies [50].

Zhang et al. developed a multilayer nanofiber mat by layering Poly-l-lactic acid with the drugs oxaliplatin and dichloroacetate. When subjected to tests, these nanofibers mats showed that they can be used as a time-programmed drug carrier in local chemotherapy against malignancies alone or in combination with already used treatment regimens, particularly for patients undergoing total tumor resection or cyto-reductive surgery. For 30 days, this multilayer device showed a synergistic impact between the two drugs and a decrease in toxicity to neighboring healthy tissues [51].

Li et al. developed a nanofibrous delivery system for dual photothermal therapy and chemotherapy. This system consisted of zwitterionic poly(2-methacryloyloxyethyl phosphorylcholine)-b-poly(ε-caprolactone) encapsulated with indocyanine green (ICG) and doxorubicin (DOX), triggered by near-infrared (NIR). It manages to convert light into thermal energy (raises the temperature by 45 °C) and, simultaneously, accelerates the release of encapsulated DOX, due to the softening of the nanofibers. This means that drug release can be controlled and turned on/off by flashing light. In addition, it is to increase cell lethality [52].

**Figure 2:**
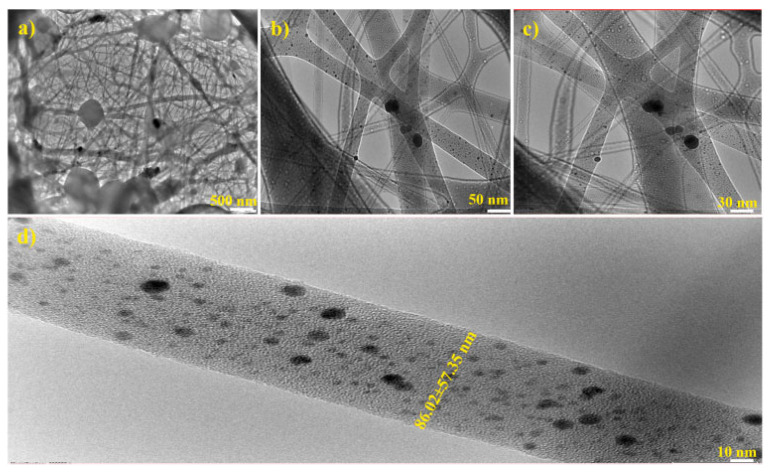
TEM images of fabricated CA/SF/Au-Ag composite NFs at different magnifications. (**a**) 500 nm; (**b**) 50 nm; (**c**) 30 nm; (**d**) 10 nm. Reprinted/adapted with permission from Ref. [53]. 2021, Elsevier.

Arumugam et al. manufactured silk fibroin/cellulose acetate/gold-silver (CA/SF/Au-Ag) nanoparticles composite nanofiber. Silk fibroin and cellulose acetate were used as reductor agent to stabilize Ag^+^ and Au^+^ anions. Figure 2 displays the TEM images of CA/SF/AuAg composite NF with different magnifications (500 nm, 50 nm, 30 nm and 10 nm). The CA/SF polymeric matrix was formed into needle and rod-shaped morphology with a range of 86.02 ± 57.35 nm in diameter. The Au and Ag nanoparticles were incorporated into the fiber matrix with an average size of 17.32 nm and 53.21 nm, respectively. Biological tests of these nanofibers were performed on breast cancer cell lines, showing excellent anticancer activity [53].

**Figure 3:**
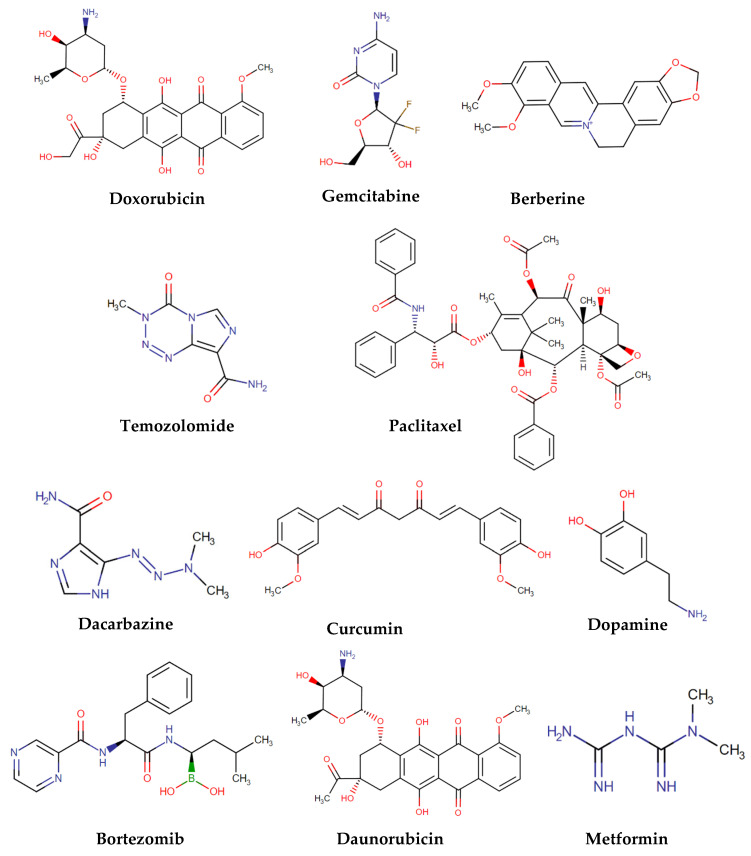
Chemical structures of the released drugs in drug delivery systems [80].

In Table 1, several examples of drug delivery systems used in cancer therapy are represented. These systems will be addressed in the following sections and have been categorized according to the nanofibers used. In Figure 3 are represented the chemical structures of the released drugs.

**Table 1:** Drug delivery systems for cancer treatment.

Fiber	Nanoparticle	Released Drug	Type of Cancer	Ref.
CH	Silver	----	----	[54]
CH-PVA	----	DOX	Ovarian	[55]
CH	----	Gemcitabine	Pancreatic	[56]
CH	----	Berberine	Cervical	[57]
CH-PVA	----	HAp/DOX	Bone cancer	[58]
CH-PU	MIL-53	Temozolomide and PTX	---	[59]
PVA-Silk	----	DOX	Breast	[60]
PVA	----	Dacarbazine	Glioblastoma	[61]
PCL-PVA	----	PTX	Colon	[62]
PLGA	----	PTX	Brain	[63]
PLGA	Gold	DOX	----	[64]
PLGA	MSNs	Curcumin	Breast	[65]
PCL-PLGA	----	DOX	----	[66]
Poly(NIPAAm-co-HMAAm	MNPs	DOX	Melanoma	[67]
CH	Fe_3_O_4_	----	----	[68]
PLGA	MIONs	Dopamine and bortezomib	----	[69]
PLA	MNPs	Daunorubicin	Leukemia	[70]
PCL	MIONs and carbogenic nanodots	DOX	----	[71]
PCL	MNPs	PTX	---	[72]
Cellulose acetate	Fe_3_O_4_	----	----	[73]
PCL	Fe_3_O_4_	DOX	---	[74]
PCL	Fe_3_O_4_	---	---	[75]
Cellulose	MNPs	DOX	----	[76]
PCL	MNPs	DOX and 17-allylamino-17-desmethoxygeldanamycin	----	[77]
*N*-isopropylacrylamide and *N*-hydroxymethylacrylamide	MNPs	Metformin	Skin melanoma	[78]
PCL	MNPs	DOX	----	[79]

CH—chitosan; PVA—polyvinyl alcohol; DOX—doxorubicin; HAp—hydroxyapatite; PTX—paclitaxel; PCL—polycaprolactone; MNPs—magnetic nanoparticles; PLGA—poly(lactic-co-glycolic acid); MIONs—magnetic iron oxide nanoparticles.

#### 4.1.1. Chitosan Fibrous Structures as Localized Drug Delivery Systems for Cancer Treatment

Chitosan (CH) is a cationic polysaccharide derived from natural chitin. CH is a biopolymer widely used in biomedical applications due to its biocompatibility, biodegradability, non-toxicity and water-solubility [34,81,82]. It is easily manufactured into different forms, namely as nanofibers [81,82].

Sanpui et al. developed a silver nanoparticle–CH nanocarrier and tested its cytotoxic effect on colon cancer cell lines. After treatment with this nanocarrier, cells were examined by fluorescence and scanning electron microscopy (morphological) and cell viability assay and flow cytometry (biochemical) to verify whether cell apoptosis had occurred. It was concluded that the use of low concentrations (24–48 µg mL^−1^) of silver nanoparticles-CH nanocarriers induced cell apoptosis, indicating its potential for use in cancer therapy [54].

**Figure 4:**
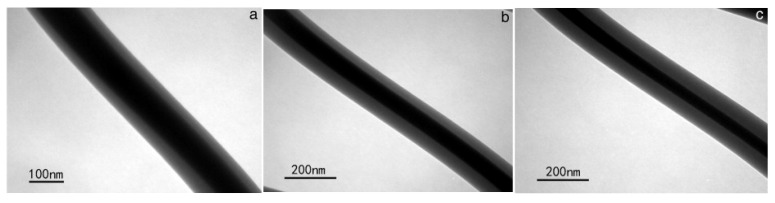
TEM images of PVA/CH core–shell composite nanofibers with different feed ratios of (**a**) 1:1; (**b**) 1:1.3; (**c**) 1:1.6. Reprinted/adapted with permission from Ref. [55]. 2014, Elsevier.

Yan et al. successfully manufactured poly(vinyl alcohol) (PVA) and CH nanofibers. The surface morphology and microstructures of the nanofibers were being altered by changing the proportion of feed between PVA and CH. In Figure 4 are represented TEM images of PVA/CH core—shell composite nanofibers with different feed ratios of 1:1, 1:1.3 and 1:1.6. The interface of the core and shell layers is clearly visible and no overlap is seen. There was a high contrast difference between the core and shell because the core and shell components possessed different densities [55].

These fibers were used as a carrier for DOX delivery, a drug used to fight cancer cells, onto human ovary cancer cells. Through observation by confocal laser scanning microscopy, it was confirmed that the prepared fibers exhibited a controlled release of DOX in the cancer cell nucleus, which were effective in prohibiting the adhesion and proliferation of ovarian cancer cells, a very important step to tumor therapy [55].

Wade et al. tested that CH-based drug-loaded fibers could be used as a device capable of locally delivering sustained high concentrations of gemcitabine. This drug is used in the treatment of cancer, with minimal toxicity for localized therapy of pancreatic cancer [56].

Jafari et al. recognized the effect of CH/Poly(Ethylene Oxide) (PEO)/Berberine (BBR) nanofibers on cancer cell lines. An inverted microscope was used to examine the development and proliferation of human breast cancer cell lines, human HeLa cervical cancer cells, and fibroblast cells in cultured media. By comparison with control group cell lines, nanofibers containing BBR concentrations of 0.5–20% by weight reduced cell proliferation. Cancer cell lines’ viability was also drastically reduced after being exposed to CH/PEO/BBR nanofibers [57].

Qavamnia et al. introduced DOX-hydroxyapatite in CH/PVA/Polyurethane nanofibers. The potential of produced nanofibers was evaluated for controlled release of DOX-hydroxyapatite and bone cancer treatment in vitro. The DOX-hydroxyapatite encapsulation efficiency on fibers was higher than 90% and its sustained release was obtained within 10 days under acidic and physiological pH. The cell attachment and cell death results also indicated the great potential of these loaded fibers for bone cancer treatment [58].

In another study, Bazzazzadeh et al. manufactured an MIL-53 nanometal organic structure combined with CH/polyurethane nanofibers grafted with poly(acrylic acid). In this structure, the drugs temozolomide and paclitaxel (PTX) were applied for the release for testing against glioblastoma cancer cells. Synthetized core-shell nanofibers had a yield superior to 80% regarding the encapsulation effectiveness of temozolomide and PTX, implying that they are potential drug carriers’ biomaterials [59].

#### 4.1.2. Polyvinyl Alcohol Fibrous Structures as Localized Drug Delivery Systems for Cancer Treatment

PVA is a Food and Drug Administration (FDA)-approved polymer with many applications for drug delivery and biomedical applications due to its physical and chemical properties [60,61]. This polymer is easy to process and produce spin, it is soluble in water, non-toxic, biodegradable and biocompatible, which makes it very interesting to be inserted into nanoparticles [61,83]. PVA fibrous structures as localized drug delivery systems are presented below.

Cao et al. fabricated PVA/silk fibroin (SF) nanoparticles with distinct core-shell structures using a coaxial electrospray technology. DOX, an anticancer drug, was created in this system, with a drug encapsulation efficiency of over 90%. By changing the PVA concentration (0.1, 0.3, and 0.5wt%), the drug’s controlled release profiles were studied. DOX was released slowly and steadily due to the barriers of carrier polymers, but its release can be accelerated using ultrasound treatment. The researchers also studied drug release in response to pH. The cell apoptosis assay showed that the sustained release of DOX increases with time and showed high cytotoxicity for breast cancer tumor cells [60].

Steffens et al. used PVA to develop a nanofibrous system based on encapsulated dacarbazine (an anticancer drug) for the treatment of recurrent glioblastoma. The produced nanofibres demonstrated 83.9 ± 6.5% drug loading, good stability and mechanical characteristics, and prolonged drug release. This regulated release improved anticancer effects such as DNA damage and cell death via apoptosis showing a system with great potential as a drug delivery system for cancer therapy [61].

**Figure 5:**
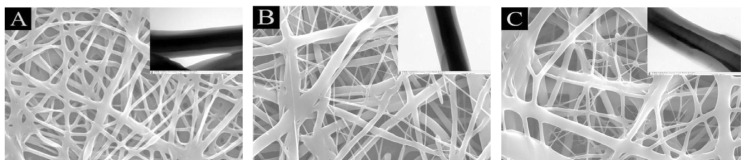
SEM images of the PCL/PVA core-shell nanofibers with flow ratios of: (**A**) 0.5:0.5; (**B**) 0.5:0.6; (**C**) 0.5:0.7; insets of the SEM images were the corresponding TEM images of the fibers. Reprinted/adapted with permission from Ref. [62]. 2021, Elsevier.

Yan et al. used polycaprolactone (PCL)/PVA nanofibres with pH-responsive properties to test as carriers of an anticancer drug, PTX. Figure 5 shows the SEM (scanning electron microscope) and TEM (transmission electron microscopy) images for PCL/PVA fibers. The flow ratio between the core and shell solutions (PCL/PVA) was 0.5:0.5, 0.5:0.6 and 0.5:0.7, respectively. Good adhesion between the fibers is visible. It has been shown that these fibers, in response to pH, release the drug. These fibers have been tested in colon cancer cells; they have shown that they can completely inhibit the proliferation and growth of cancer cells and can even cause their death. This indicates that they have promising abilities to be used as biomaterials in the therapy of some tumors [62].

#### 4.1.3. Poly(lactic-co-glycolic acid) Fibrous Structures as Localized Drug Delivery Systems for Cancer Treatment

Poly(lactic-co-glycolic acid) (PLGA) is a polymeric nanoparticle approved by the FDA for use in drug delivery systems, due to its properties of controlled and sustained release, low toxicity, effective biodegradability and biocompatibility with tissues and cells. This polymer undergoes hydrolysis in the body producing monomers of biodegradable metabolites, lactic acid and glycolic acid. This results in very low toxicity because the human body is able to metabolize these monomers through the Krebs cycle [84]. Below are some examples of localized drug delivery systems using PLGA fibers.

Xie et al. manufactured, by electrospun, micro, and nanofibers based on PLGA as implants for the treatment of brain cancer. These PLGA-based micro and nanofibers were encapsulated with the drug PTX, with an encapsulation efficiency greater than 90%. Sustained drug release was achieved for more than 60 days and toxicity test results showed an IC_50_ value of PLGA nanofibers with PTX comparable to PTX alone [63].

**Figure 6:**
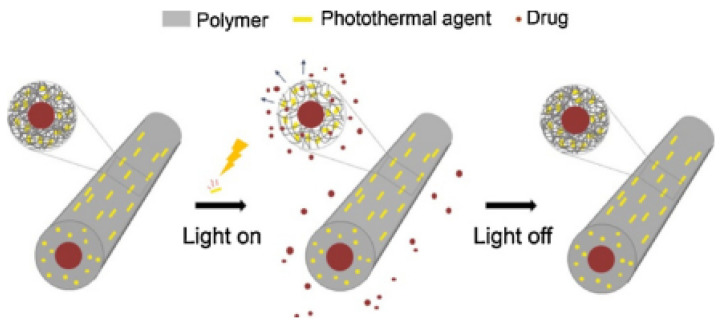
Working principle for a fibrous system capable of NIR light-sensitive, on-demand release of a drug. Reprinted/adapted with permission from Ref. [64]. 2019, Elsevier.

Choi et al. employed PLGA to allow hollow fibers’ production. NIR light sensitivity was incorporated with a segmental switch ability in its chain for cancer therapy. These fibers were responsible for providing a core that encapsulated DOX and a shell that entrapped gold nanorods as a photothermal agent. On exposure to NIR light, the photothermal agent generated heat to increase the local temperature of the fibers, which depended on the power density of the NIR light. As the temperature was above the glass transition of the polymer, the PLGA chains became mobile. This increased the free volume within the shell which led to rapid drug release. When the NIR light was turned off, heat generation was interrupted by the inactivation of the photothermal agent and froze the segmental movement of the chains, interrupting drug release. Figure 6 shows an operating principle for a fibrous system made of a polymer and an NIR light-absorbing photothermal agent [64].

Regarding cell viability, when not exposed to NIR light, cell viability above 90% was observed, proving excellent biocompatibility. With six cycles of NIR light exposition, the cell viability decreased to 8%, proving that most of the cells were dead and that the system could properly work as an anti-cancer one [64].

**Figure 7:**
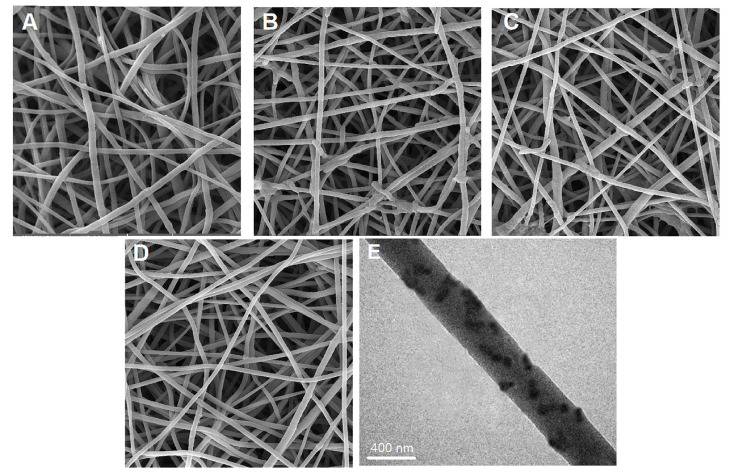
Morphology of electrospun NFs. FE-SEM images of: (**A**) neat PLGA NFs; (**B**) MSNs/PLGA NFs; (**C**) CUR/PLGA NFs; (**D**) CUR@MSNs/PLGA NFs. (**E**) TEM image of CUR@MSNs/PLGA NFs. Reprinted/adapted with permission from Ref. [65]. 2021, Elsevier.

In another study, Mohebian et al. incorporated curcumin (a natural antitumor agent) into mesoporous silica nanoparticles (MSNs), and these were, consequently, incorporated into PLGA, originating an electrospun nanofiber-mediated drug release system (CUR@MSNs/PLGA nanofibers). The morphology of electrospun NFs (neat PLGA NFs, MSNs/PLGA NFs, CUR/PLGA NFs, and CUR@MSNs/PLGA NFs) was studied by SEM and TEM and is depicted in Figure 7. The average diameter of the MSNs/PLGA NFs and CUR@MSNs/PLGA NFs were found to be (600 ± 125 and 620 ± 144 nm, respectively) which were greater than neat PLGA NFs and CUR/PLGA NFs (480 ± 78 nm and 510 ± 150, respectively), owing to the increment in the viscosity of the mixed solution with the content of mounting MSNs. The TEM image shows us the curcumin incorporated in the fibers. This system was tested in breast cancer and the results showed that the CUR@MSNs were successfully incorporated into the PLGA nanofibers, exhibiting a sustained and prolonged drug release profile. This composite nanofiber also had greater in vitro cytotoxicity, low migration, and was capable of enhancing apoptosis induction, thus being a promising application for the treatment of cancer [65].

Khanom et al. created a single multifunctional PCL-PLGA-DOX nanofiber mats-using pyrroleon with in situ polymerization on the surface. Pyrrole is a powerful photothermal agent that, through absorption of NIR energy, can increase the temperature of the surrounding medium at various concentrations. In response to an 808 nm NIR laser, the pyrrole-coated fiber mats demonstrated outstanding photothermal conversion capability and hyperthermia. The pyrrole-coated PCL-PLGA-DOX membrane also had a better inhibitory impact than non-irradiated mats or mats that only caused hyperthermia. A possible justification is the greater release of DOX at the site due to fiber mobility and the hyperthermia effect. These results confirm the promising application of these nanofibers as a localized drug delivery cancer treatment [66].

#### 4.1.4. Magnetic NPs Combined with Fibrous Structures as Localized Drug Delivery Systems for Cancer Treatment

Magnetic nanoparticles (MNPs) are widely utilized nanomaterials with enormous potential in various biomedical applications. These include controlled drug release [85,86,87,88], hyperthermia, magnetic resonance imaging diagnosis (MRI) [88,89], gene therapy, and regenerative medicine [90].

The inherent magnetism of these materials facilitates targeting and therefore they are a viable option in smart drug delivery systems. These nanoparticles will act as drug carriers that will be directed through an external magnetic field to the desired location, where the drug will be released. To speed up the process of delivering the medicine in time and to the specific target, it is common to resort to inducing an alternating magnetic field (AMF) leading to the movement of nanoparticles [91].

As described so far, the fibers have a potential application for local drug delivery. Their combination with MNPs may further increase their interest for cancer treatment [92]. Below are described several drug delivery systems using MNPs combined with fibers.

**Figure 8:**
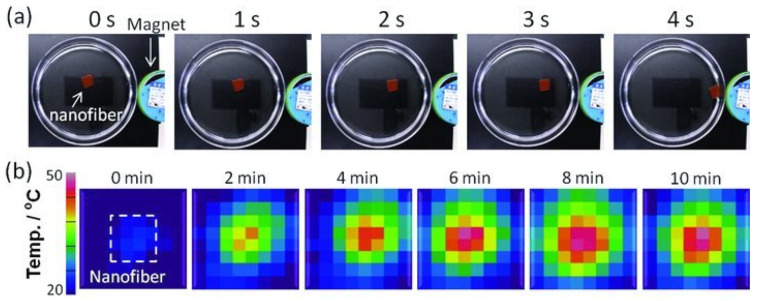
(**a**) Magnet-responsive behaviour of DOX/MNPs-nanofibres. (**b**) Time-dependent infrared thermal images of the DOX/MNPs-nanofibres in an AMF. Reprinted/adapted with permission from Ref. [67]. 2022, John Wiley and Sons.

Kim et al. developed a smart hyperthermia nanofiber with simultaneous heat generation and drug release in response to “on-off” switching of the AMF. The nanofiber was composed of chemically cross-linkable temperature-responsive polymer (poly(NIPAAm-co-HMAAm), DOX, and MNPs. To study the response of nanofibers to an AMF, a neodymium magnet was kept near a dish in which MNPs-nanofibers were used, as shown in Figure 8a. In 5 s, the nanofibers were attracted by the magnet, showing their response behavior and the potential for manipulation under a controlled magnetic field. In Figure 8b are infrared thermal images of nanofibers (25 mg/300 μL, after crosslinking) in an AMF application with 31 wt% MNPs. The temperature rises in the middle of the photos where the nanofibers are placed, indicating that MNPs-nanofibers might be employed to treat hyperthermia. Even in the presence of this device, 70% of human melanoma cells died after just 5 min of application of an AMF, due to the double effect of heat and drugs, again showing the immense potential of this area [67].

Lin et al. used Fe_3_O_4_ NPs incorporated onto crosslinked electrospun CH nanofibres using chemical coprecipitation. Iminodiacetic acid was also grafted in CH to increase the amount of MNPs formed in the magnetic nanofiber composite. This incorporation led to the formation of more MNPs in the nanofiber matrix. In addition, the magnetic IDA-grafted CH nanofibers composite showed that it could reduce the proliferation/growth rate of malignant cells of the tumor under the application of magnetic field. This system can be delivered to the treatment site precisely by surgical or endoscopic method [68].

**Figure 9:**
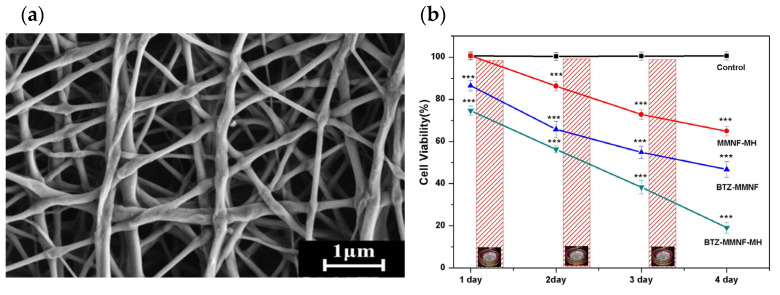
(**a**) FESEM image of electrospun PLGA nanofibres and MNF; (**b**) Anticancer effects of MMNF-MH (hyperthermia alone), BTZ-MMNF (drug alone) and BTZ-MMNF-MH (hyperthermia with drug-loaded magnetic nanofibre) on 4T1 cell lines under repeated application of hyperthermia on days 1, 2, and 3. (Asterisks indicate a significance in toxicity using ANOVA test [*** *p* < 0.001]) Reprinted/adapted with permission from Ref. [69]. 2016, Elsevier.

Sasikala et al., reported a smart nanoplatform responsive to a magnetic field to administer both magnetic hyperthermia (MH) and pH-dependent anticancer drug release for cancer treatment. For this, magnetic iron oxide nanoparticles (MIONs) were incorporated into the nanofiber matrix, forming a magnetic nanofiber matrix (MMNF). To develop this nanofiber, PLGA was used (Figure 9a). Regarding the anticancer drug delivery, this step was realized by surface functionalization using dopamine to conjugate the bortezomib through a catechol metal binding in a pH-sensitive manner. The in vitro studies verified that the device demonstrated a synergistic anticancer effect by applying hyperthermia and drug delivery simultaneously. This approach provides a secure route for targeted anticancer drug delivery to the tumor, as evidenced by Figure 9b, and ensures that the MNPs are sufficiently concentrated in the tumor region to enable hyperthermia treatment [69].

For a treatment of leukemia cancer, Hosseini et al. used electrospun polylactic acid (PLA) nanofibers incorporated with MNPs and multiwalled carbon nanotubes (MWCNT). As a model drug, they chose daunorubicin, which was successfully encapsulated in the synthesized nanofibrous scaffolds. They also investigated the release rate and cell proliferation of K562 cancer cells, and it was clear that the presence of a magnetic field increased both of these metrics. This applied magnetic field also reduced cell viability, increasing the inhibition effect on K562 cancer cells, indicating its synergistic effect and promising efficacious behavior [70].

Tiwari et al. created a magnetically actuated smart-textured fibrous system based on PCL with MIONs, DOX and fluorescent carbogenic nanodots. By applying an AMF, the system demonstrated enhanced heating, showing that more than 90% of HeLa cells were dead by apoptotic-necrotic mechanism. The use of AMF also accelerated the release of the drug and increased the effectiveness of the therapy, evidencing its ability to navigate in the fluid. This system also proved to be non-toxic to the cells and during incubation it also did not release toxic materials, proving to be a good option in the treatment of cancer [71].

**Figure 10:**
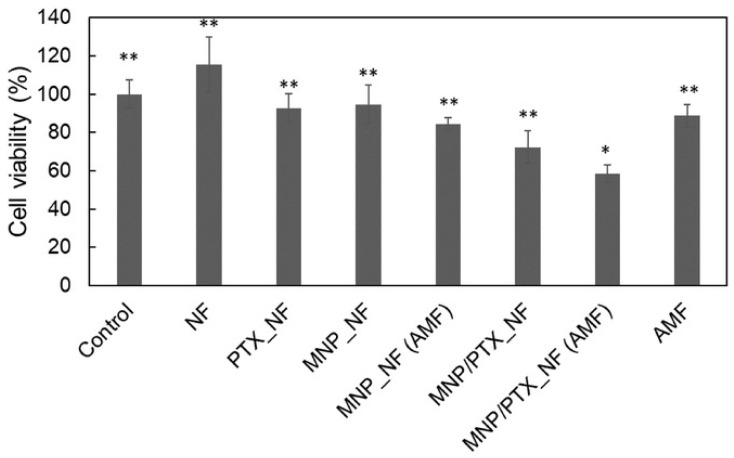
Cytotoxicity test using NF (nanofibre mesh without MNPs and PTX), MNP_NF (nanofibre mesh with only MNPs), MNP_NF (AMF) (nanofibres mesh with only MNPs upon AMF), PTX_NF (nanofibres mesh with 0.3 mg of PTX), MNP/PTX_NF (AMF) (nanofibres mesh with MNPs and 0.3 mg of PTX upon AMF) and AMF (*, ** *p* < 0.05). Reprinted/adapted with permission from Ref. [72]. 2022, John Wiley and Sons.

Also using PCL, Niiyama et al. developed a nanofibrous mesh incubated with an anticancer agent, PTX, and MNPs. In vitro tests showed that the drug was released slowly over six weeks. Furthermore, when the mesh was excited with an AMF, the MNPs within the nanofibers created localized heat, which promoted heat-induced cell death as well as enhanced chemotherapeutic impact of PTX. This was also confirmed with a cytotoxic test, where heating and the release of PTX were combined, and 58% of tumor-bearing mouse cells (NCI-H23 cells) died (Figure 10) [72].

Matos et al. mixed MNPs (Fe_3_O_4_) with cellulose acetate to form composite membranes for MH. To create stable suspensions at physiological pH, the supermagnetic NPs were stabilized by oleic acid (OA) or dimercaptosuccinic acid (DMSA). Through SEM and TEM its incorporation into the fiber matrix was confirmed. It was feasible to obtain therapeutic temperatures by adjusting the quantity of Fe_3_O_4_ NPs present on cellulose acetate mats and modelling the parameters of the hyperthermia experiment. The tensile studies confirmed that the addition of these NPs had a considerable influence on the mechanical response of cellulose acetate, raising Young’s Modulus, elastic limit stress, and ultimate tensile strength. In vitro research has shown that the concentration of Fe_3_O_4_ NPs in these membranes must be regulated so as not to exceed the clinically necessary heat, to avoid cytotoxic effects that could harm healthy tissues. Composite fibers’ membranes with included DMSA-Fe_3_O_4_ NPs proved to be the most promising for application in MH, considering heating ability and lack of cytotoxicity, after adsorption in a solution with a concentration of 0.5 mg mL^−1^ [73].

Suneet et al. manufactured magnetic nanofibrous mat-based bandage using an external AMF-induced hyperthermia to treat skin cancer non-invasively. The authors used the electrospinning technique to manufacture the Fe_3_O_4_ nanoparticles-incorporated PCL fibers-based bandages. The efficacy of the bandage was investigated in vitro using parental/DOX hydrochloride—resistant HeLa cells and in vivo using BALB/c mouse model in the presence of an external AMF. The results showed that this system dissipates thermal energy locally in the application of external AMF and increases from room temperature to 45 °C in a controlled manner in a few minutes. It has also been found that elevated temperature can significantly kill parental and Dox-resistant HeLa cells. When fibrous mat-containing Dox was incubated with HeLa cells and exposed to an AMF for 10 min, more than 85% of the parental HeLa cells were killed, likely due to the enhanced activity of Dox at higher temperatures. In vivo tests confirmed the full recovery of chemically induced skin tumors on BALB/c mice within a month after five hyperthermic doses for 15 min. There were also no signs of post-therapy inflammation and cancer recurrence [74].

Hu et al. also created a drug delivery system with NPs, using PCL and Fe_3_O_4_. Fiber sizes ranged from 4 to 17 µm, due to the different percentages of NPs that were tested in the fibers. The magnetic composite fiber membranes showed excellent heating efficiency and thermal cycling characteristics. To prove this, the temperature increase of the fibers with different percentages of MNPs was studied and is represented in Table 2. The heating temperature increased rapidly over time and eventually stabilized after reaching a certain temperature. Starting from an initial temperature of 15 ℃, it was observed that the temperature increased with the concentration of MNPs in the fibers following the application of an AMF. These results reveal that PCL/Fe_3_O_4_ fiber membrane has the potential to be used in the treatment of hyperthermia [75].

Also from cellulose nanofibers and MNPs, Sumith et al. developed a pH-responsive and bioactive DOX delivery system. This device had a saturation magnetization of 50.1 emu g^−1^ and a strong drug cellular internalization index. This system exhibits strong hyperthermia potential in an AMF and pH-triggered DOX delivery. Measuring the in vitro cytotoxicity as a function of DOX release from the samples confirmed its efficacy. With these advantageous multifunctional activities, it has been demonstrated that this system can be successfully recommended for cancer treatment applications, namely hyperthermia [76].

**Figure 11:**
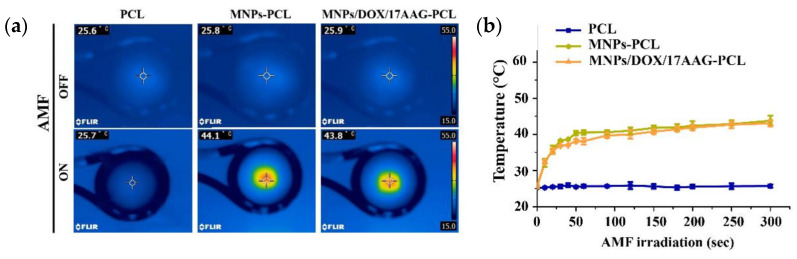
(**a**) PCL-based nanofibers’ mesh infrared thermal imaging; (**b**) PCL-based nanofibers’ mesh with different contents during AMF irradiation at different times. Reprinted/adapted with permission from Ref. [77]. 2021, International Journal of Molecular Sciences.

Chen et al. manufactured a magnetic composite nanofiber mesh using PCL with DOX, MNPs and 17-allylamino-17-desmethoxygeldanamycin. This system can achieve mutual synergy of hyperthermia, chemotherapy and thermomolecular-targeted therapy for highly potent therapeutic effects. The developed nanofiber mesh exhibits hyperthermia, good biocompatibility, a sustained release behavior and is pH-sensitive. These features are favorable for long-term maintenance of effective drug concentration in tumor tissue. As it can be seen by Figure 11a,b, infrared thermal images of PCL, MNP-PCL, and MNP/DOX/17-allylamino-17-demethoxygeldanamycin (17AAG)/PCL nanofiber meshes loaded with 12.0 mg of MNPs’ AMF irradiation lead to the conclusions that the temperature of an AMF-exposed mesh of MNP-PCL and MNP/DOX/17AAG-PCL MNPs increased from 25.8 °C and 25.9 °C to 44.1 °C and 43.8 °C, respectively, while in the PCL nanofiber mesh there were no significant changes in temperature. In MCF-7 breast cancer cell lines, this nanofiber mesh efficiently induced apoptosis, demonstrating its potential as a new tumor therapy and as an effective locally implantable system to enhance the effectiveness of cancer combination therapy [77].

To develop a smart hyperthermia nanofibrous scaffold, Samadzadeh et al. used temperature responsive polymers (*N*-isopropylacrylamide and *N*-hydroxymethylacrylamide) blended with MNPs (10 nm) and mesoporous silica NPs loaded with metformin. This system is capable of generating heat and releasing metformin in two stages in response to the “on-off” switching of an AMF for better hyperthermic chemotherapy. Tests were performed to study the rate of swelling with reversible changes and the associated drug discharge in response to an AMF application with on-off switching. It was found that when applying an AMF for 300 s, during the second and third days, the metabolic activity of B16-F10 skin melanoma cells incubated with the system was decreased. The resultant system also demonstrated a persistent release, showing a great combination of early quick and late extended drug discharge as intended [78].

**Figure 12:**
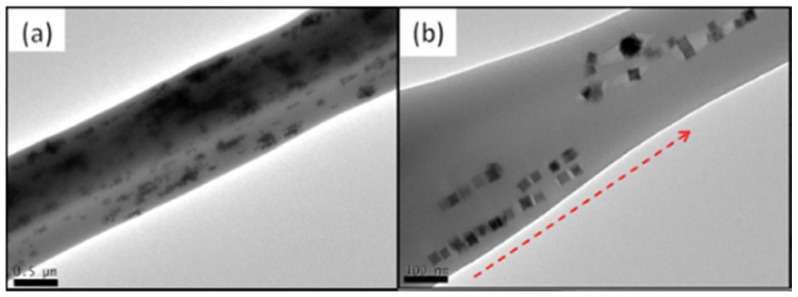
TEM images of the DOX-loaded magnetic fibers. (**a**)-Images of PCL-MNPs-DOX fibers. In (**b**) the spontaneous alignment (red arrow) along the fiber axis can be observed. Reprinted/adapted with permission from Ref. [79]. 2022, Elsevier.

More recently, Serio et al. presented the design, fabrication and characterization of biocompatible fibers of PCL co-loaded with DOX as well as MNPs of cubic shape. The co-loading of DOX within the magnetic fibers was made possible by simply adding the drug to the solutions containing the PCL polymer and the MNPs. These fibers were obtained with 0.5–1 mm in diameter. Its characterization was done by TEM analysis (Figure 12) and proved the incorporation of nanocubes inside the fibers. Also visible is the preferential alignment of the nanocubes into small chains within the length of the fiber [79].

When compared to individual treatments (only non-specific drug release and only MH without drug load), the heat induced by MH combined with DOX release led to increased mortality (cell viability < 20%) in the group of cells treated with PCL-MNPs-DOX and subjected to MH therapy [79].

In conclusion, as it can be seen from the review of all these articles, fibrous structures are capable of acting as drug delivery systems for the treatment of various cancers.

### 4.2. Nanofibers Theranostic Drug Delivery Systems for Cancer Treatment

The term “theranostics” is used when a treatment incorporates therapy and medical imaging personalized treatment for individual patients, allowing a more informed monitorization of the substance into the patient and its effects. Fibers have gained attention as a promising platform for theranostic drug delivery systems due to their high surface area for drug loading, tunable release rates, and localized delivery. Additionally, fibers can be engineered to incorporate imaging agents, enabling simultaneous disease diagnosis and treatment. With their versatility and biocompatibility, fibers have a high potential for the development of novel theranostic delivery systems for various diseases [12].

Extensive research has been conducted to explore the therapeutic properties of nanoparticles in the previous sections. Therefore, this section of the review will focus more on the diagnostic properties of nanoparticles, specifically their use in imaging and sensing applications. By utilizing the unique properties of nanoparticles, such as their size, shape, and surface chemistry, researchers have been able to develop highly sensitive and specific imaging and sensing platforms. These platforms have shown great potential in the detection and diagnosis of diseases, as well as the monitoring of treatment response.

There are several types of fibers that have been explored for use in theranostic drug delivery systems, including polymeric fibers, inorganic fibers, and natural fibers [12,93].

As established before, polymeric fibers are biocompatible and biodegradable, making them an attractive option for drug delivery. Furthermore, as we will see, it is possible to observe that these particles can also be functionalized with imaging agents for theranostic applications, making them ideal candidates for these functions.

Soares et al. created a drug delivery system utilizing PLGA nanofibers that integrated contrast agents for MRI. This system included superparamagnetic nanoparticles which enhance the nuclear relaxation of water protons, resulting in a decrease in contrast (darker contrast) in the transverse (T2) relaxation. Therefore, the inclusion of MNPs facilitates treatment monitoring through MRI [94].

Liao et al. developed an NP system in which a PLGA core was coated using a paramagnetic substance. Similar to the previous study, this approach also allowed drug delivery through PLGA and monitorization through MRI due to the surface properties. Not only this, due to the coating, the treatment with the aforementioned method resulted in improved cellular internalization compared to untreated nanoparticles, potentially reducing chemotherapy side effects and facilitating targeted delivery of anti-cancer drugs [95].

**Figure 13:**
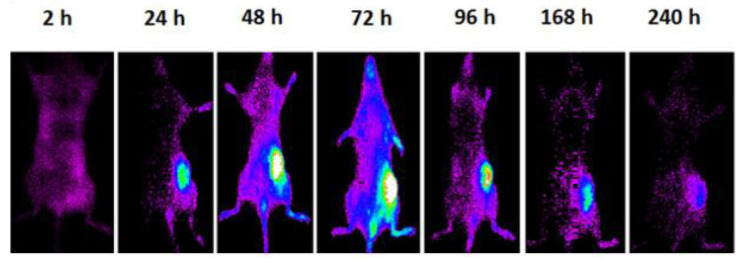
The mouse with a subcutaneous syngeneic tumor was imaged using whole-body optical imaging at various time points (2, 24, 48, 72, 96, 168, and 240 h) after the local administration of 500 µg of fluorescent PLGA-NPs subcutaneously. Reprinted/adapted with permission from Ref [96]. 2020, Journal of Clinical Medicine.

In the study by Varani et al., specific PLGA-NPs were chosen and modified with a NIR fluorochrome, enabling fluorescence to penetrate deeper into tissues. It is possible to observe in Figure 13 how the particles acted as both a drug carrier and an imaging agent [96]. A similar approach was taken by Park et al., where PCL fibers were loaded with surface openings containing biocompatible photothermal agent. Even though this agent could be used for imaging purposes, in this case it was adapted to be used for triggered release of the drug through NIR Light [97].

Inorganic fibers also have potential as drug delivery platforms due to their high surface area and tunability, as previously observed. Various shapes and sizes of particles can be synthesized, and for specific applications, imaging moieties can be attached to them, as we will see next.

As Jafari et al. highlighted, the fabrication of implants has gained increasing attention due to the nontoxic and nanotopographical characteristics of TiO_2_ nanomaterials. Unlike polymeric fibers, these materials by themselves have imaging properties since it is possible to use them as biosensors through two main methods: excitation (light) and detection (current). With these methods, it is able to give highly accurate readings with a good performance and low noise interference, significantly improving the detection of targets. The article discusses the limitations of using TiO_2_ nanomaterials in biomedical applications, including their inability to absorb a significant portion of the solar spectrum and the potential damage to biomolecules caused by photo-generated holes. It also emphasizes the importance of investigating the toxicity of TiO_2_ nanostructures in various biomedical applications, such as drug delivery and implants [98].

Natural fibers are biocompatible, biodegradable, and abundant in nature, making them an attractive option for drug delivery. They can be functionalized with various targeting or imaging agents and can be tailored to exhibit specific properties for theranostic applications [94].

He et al. combined silk fibroin nanofibers to make a hydrogel hybrid system. It exhibited remarkable imaging characteristics using an upconversion luminescence (UCL) imaging diagnosis, a photon-emitting optical process resulting from the absorption of two or more low-energy photons [99]. The in vivo experiments showed significant inhibition of tumor growth in mice, indicating that the hydrogel system has the potential to be used for both tumor imaging and anti-tumor therapy in clinical applications [100].

Ma et al. created natural silk fibroin nanofibers and encapsulated clinical indocyanine green molecules in them to perform in vivo NIR-I/II fluorescence imaging. The coupling of these two nanomaterials was performed to inherit their safety and biocompatibility. This approach is unique as the longer wavelengths of the light provide fast feedback and high resolution at the cost that these particles have a relatively low lifetime [101].

Overall, the choice of fiber material for theranostic drug delivery depends on several factors, such as the type of disease being targeted, the drug being delivered, and the desired release profile. Each type of fiber has its own unique properties and advantages that make it suitable for specific applications.

Incorporation of imaging agents is a crucial aspect of the development of theranostic drug delivery systems based on fibers as it can help to visualize the drug delivery process and monitor the fate of the drug within the body [102,103]. Two common types of imaging agents used in theranostic drug delivery systems, as shown before, are fluorescent dyes and MNPs.

Fluorescent dyes are commonly used for optical imaging of the drug delivery process. These dyes emit light when excited by a specific wavelength of light, and the intensity of the emitted light can be used to determine the concentration and location of the dye. The incorporation of fluorescent dyes into fibers allows for real-time visualization of the drug delivery process and can provide valuable information about the distribution and pharmacokinetics of the drug [104,105]. However, although fluorescent probes are useful in pre-clinical applications, they have limited tissue penetration and are not appropriate for human studies. On the other hand, the limited light penetration can be overcome through the use of radioactive isotopes, including Copper-64 for positron emission tomography (PET) or Technetium-99m for gamma-camera imaging. Functionalizing with targeting molecules, such as VEGF, may mitigate the serious problem of liver and kidney radiotoxicity posed by the use of radioisotopes, particularly alpha or beta emitters [96].

MNPs are commonly used for MRI of the drug delivery process. These nanoparticles have magnetic properties that allow them to interact with a MNO field and produce a signal that can be detected by an MRI scanner. The incorporation of MNPs into fibers allows for real-time monitoring of the drug delivery process and can provide information about the biodistribution and pharmacokinetics of the drug [106,107]. Another advantage of this imaging technique is that, depending on the material used, it might not be necessary to add any additional agent, as it could be seen when using inorganic fibers, such as TiO_2_ described above [98]. 

The field of diagnostic nanofibers is rapidly advancing and holds great promise for the future. Theranostic nanocarriers offer an additional feature when compared to regular delivery systems, which is the ability to track their localization in the body and provide information on the progression of the disease and the efficacy of the therapy [108]. 

## 5. Conclusions

Cancer is one of the main causes of death worldwide and despite intensive research in this area and numerous strategies developed to treat this disease, there is still no ideal solution to cure this disease. There are several treatments available for cancer, such as surgery, chemotherapy, radiotherapy, immunotherapy, endocrine therapy, photodynamic therapy and hyperthermia therapy, but they all have significant drawbacks, making the treatment dangerous and very painful for the patient. For this reason, research continues to be carried out, looking for alternatives that are better than the previous ones.

Drug delivery systems have been intensively studied, their objective being to reach only the necessary target, preserving healthy cells, reducing the side effects of patients. These are technologies designed to deliver medicinal substances in a targeted and/or regulated manner. Several structures can be used as polymeric drug delivery systems, such as pharmacological films, hydrogels, wafers, sticks, microspheres, and fibers, among others.

Fibers (micro and nanofibers) can create very promising material structures for localized drug delivery systems, especially regarding cancer treatment. The properties of fibers can be affected by the materials used to produce them. Biodegradable and biocompatible polymers, such as CH, PVA, PLGA, PCL, MNPs, and others, have been utilized to achieve the desired application while minimizing the therapy’s risk.

As demonstrated, numerous studies have indicated that the incorporation of nanoparticles into fibrous substrates yields promising outcomes, imparting a diverse range of properties and reducing the proliferation and growth rate of cancerous cells in the tumor. Fibrous structures can also be used in theranostics, that is, in the detection and diagnosis of the disease, in addition to monitoring the response to treatment. It is also possible to incorporate imaging agents, allowing the diagnosis and treatment of diseases. Despite the progress made, none of these systems have successfully passed clinical trials and are not yet available for treating patients. Therefore, they still face several challenges that must be overcome before reaching their full potential.

## Figures and Tables

**Table 2:** Temperature rise depending on the concentration (wt%) of Fe_3_O_4_ NPs. Adapted from [75].

Fe_3_O_4_ (wt%)	ΔT (°C)
5	21.0 ± 0.4
9	25.2 ± 0.3
13	27.1 ± 0.5
17	31.1 ± 0.2
21	36.4 ± 0.3

## Data Availability

Data sharing not applicable.
